# Lateral suspension vs. sacral colpopexy for treating pelvic organ prolapse: a systematic review and meta-analysis

**DOI:** 10.1007/s00404-025-08210-4

**Published:** 2025-10-03

**Authors:** Andrea Lombisani, Veronica Tius, Chiara Ferraro, Martina Arcieri, Lorenzo Vacca, Daniela Caramazza, Stefano Restaino, Tommaso Simoncini, Giampiero Capobianco, Alfredo Ercoli, Giovanni Scambia, Giuseppe Vizzielli, Giuseppe Campagna

**Affiliations:** 1 Gynecological Surgery Unit, Dipartimento Centro di Eccellenza Donna e Bambino Nascente, Ospedale Isola Tiberina-Gemelli Isola, Rome, Italy; 2 Medical Area Department (DAME), in Department of Medicine (DMED), University of Udine, Piazzale S. Maria Della Misericordia 15, Udine, Italy; 3 Università Cattolica del Sacro Cuore, Fondazione Policlinico Universitario Agostino Gemelli IRCCS, Rome, Italy; 4 Clinic of Obstetrics and Gynecology, “S. Maria Della Misericordia” University Hospital, Azienda Sanitaria Universitaria Friuli Centrale (ASUFC), Udine, Italy; 5 PhD School in Biomedical Sciences, Gender Medicine, Child and Women Health, University of Sassari, Sassari, Italy; 6 Division of Obstetrics and Gynecology, Department of Clinical and Experimental Medicine, University of Pisa, Pisa, Italy; 7 Department of Clinical and Experimental Medicine, Gynecologic and Obstetric Clinic, University of Sassari, Sassari, Italy; 8 Department of Human Pathology in Adult and Childhood “G. Barresi”, Unit of Gynecology and Obstetrics, University of Messina, Messina, Italy; 9 Department of Woman and Child’s Health and Public Health, Fondazione Policlinico Universitario Agostino Gemelli IRCCS, Rome, Italy

**Keywords:** Pelvic organ prolapse, Sacral colpopexy, Lateral suspension, Sacral suspension, Mesh, Laparoscopy, Minimally invasive surgery

## Abstract

**Purpose:**

Although sacral colpopexy is considered the gold standard for correcting apical prolapse, it is associated with extended operative times and surgical complications. An alternative surgical approach is currently being investigated. This meta-analysis aims to summarize and compare the available data on laparoscopic sacral colpopexy (LSCP) and laparoscopic lateral suspension (LLS) as per the Dubuisson technique.

**Methods:**

A systematic search of PubMed (MEDLINE) and Google Scholar was conducted from the inception of each database until December 2024. Studies comparing LSCP and LLS on at least one efficacy outcome selected. Objective or subjective success rate, surgery-related data and follow-up data were extracted. Results were pooled using a random-effect meta-analysis.

**Results:**

A total of 6 studies were included. The meta-analysis did not report statistical differences between LSCP and LLS in terms of apical prolapse [OR = 1.24; CI 95% (0.61, 2.52); *I*^2^ = 0%; *P* = 0.55] and anterior prolapse [OR = 0.78; CI 95% (0.45, 1.37); *I*^2^ = 0%; P = 0.39] correction. Subjective success rate was similar (*P* = 0.72). LLS required shorter operative time [43.1 min, CI 95% (16.75, 69.45); *I*^2^ = 97%; *P* = 0.001]. No major differences were found regarding intraoperative and early post-operative complications, re-operation and recurrence rates. Follow-up data regarding quality of life showed no significant differences about de novo stress urinary incontinence, intestinal impairment, sexual function, and pain after surgery.

**Conclusions:**

LLS provides similar outcomes to LSCP for apical and anterior prolapse in selected cases. However, limited long-term data and few studies on advanced prolapse prevent LLS from being declared an equally effective alternative at this time.

**PROSPERO registration number:**

CRD42024537270.

**Supplementary Information:**

The online version contains supplementary material available at 10.1007/s00404-025-08210-4.

## Introduction

### Background

Pelvic organ prolapse (POP) is a pathology that affects the quality of life of more than 40% of women [1]. It is estimated that 12% of the female population will undergo POP surgery at some point during their lifetime [2], posing a predictably substantial burden on the healthcare system and costs to society. The primary challenge in managing this condition is the high incidence of surgical intervention to treat recurrence. In this regard, restoring the apex of the vagina when it is involved in prolapse is essential to achieve a successful outcomes [3]. Despite being considered as the least invasive, vaginal surgery has shown lower success rates than abdominal mesh surgery [4]. Currently, sacral colpopexy (SCP) with a minimally invasive approach is held as the gold standard for the treatment of apical prolapse, demonstrating the lowest re-intervention rate. Although laparoscopic SCP (LSCP) is an effective procedure, it is associated with prolonged operative times and requires expertise in suturing and anatomical dissection. Furthermore, SCP requires access to the presacral region, which carries inherent risks of vascular and nerve damage [5]. The rationale behind the development of laparoscopic lateral suspension (LLS) with mesh was to identify a surgical technique that could be more readily reproduced, carry a lower risk profile, and offer comparable efficacy to LSCP for the management of advanced POP. Dubuisson first conceived the procedure in 1998 [6], drawing inspiration from Kapandji’s laparotomy technique [7].

### ***Objectives***

LLS has gained increasing popularity in the field of urogynecologic surgery, particularly due to its favorable outcomes while requiring less time and avoiding dissection of the sacral area. Still, only one systematic review [8] has reported promising anatomical and subjective results for this technique. We conducted a systematic review with meta-analysis comparing minimally invasive sacral colpopexy with lateral suspension, to determine whether LLS can genuinely be considered a viable alternative to SCP.

## Materials and methods

### Search strategy

To identify relevant studies to this meta-analysis, a systematic search of PubMed (MEDLINE) and Google Scholar databases was performed including the following MeSH terms: “pelvic organ prolapse”, “urogenital prolapse”, and “lateral suspension”. The full search strategy is provided in Supplemental materials (Appendix S1). The literature review covered the period from the inception of the databases up to December 1, 2024, and the articles found in the literature were uploaded onto the Rayyan platform. References from pertinent articles were manually examined to identify additional relevant studies. This systematic review and meta-analysis has been conducted following the Preferred Reporting Items for Systematic Reviews and Meta-analyses (PRISMA) [9] guidelines and registered with the International Prospective Register of Systematic Reviews (PROSPERO) (Registration number: CRD42024537270).

### Eligibility criteria and study selection

Study selection was based on pre-defined PICO (Population, Intervention, Comparison, and Outcome) criteria. Population: adult women undergoing surgical repair of pelvic organ prolapse. Intervention: minimally invasive (laparoscopic or robotic) lateral suspension employing the Dubuisson technique, with or without concomitant hysterectomy. We excluded studies enrolling patients undergoing different lateral suspension techniques other than the Dubuisson technique. Comparison: minimally invasive (laparoscopic or robotic) sacral colpopexy with or without concomitant hysterectomy. Outcome: objective and subjective success rates as primary outcomes; surgery-related data (i.e., intra- and post-operative complications, operative time, total blood loss, and length of hospital stay) and follow-up data (i.e., recurrence rate, re-operation rate, and long-term complications) as secondary outcomes of interest. Outcome eligibility required reporting at least one of the primary outcomes of interest.

Published material underwent the same rigorous methodological evaluation. Single case reports, conference proceedings, meta-analyses, book chapters, and editorial letters were excluded. No restrictions were applied regarding language or publication date. Three reviewers (AL, VT, and CF) independently reviewed and screened titles and abstracts based on the pre-defined strategy and criteria. Full texts of selected studies were then retrieved. The authors discussed discrepancies in decisions regarding study inclusion until they reached an agreement.

### Data extraction

Microsoft Excel was used to collect and summarize data. Three parallel data extractions were performed independently by three authors (AL, VT, and CF). The following data and information were extracted for each study, if available: first author, year of publication, country of origin, study design, type of surgical procedure, type of mesh used, sample size, characteristics of participants (i.e., median age, median Body Mass Index, and median parity), type of pelvic organ prolapse, advanced pelvic organ prolapse stage rate (III–IV stages), previous POP surgery rate. Subsequently, surgery-related data, such as operative, intraoperative complications (i.e., bladder, rectal, and vaginal injury) and early post-operative complications according to Clavien–Dindo classification [10], conversion rate to laparotomy, blood loss and hospital stay was extracted. Outcomes data with their definition was then collected: anatomical and subjective success rate as reported in each study, sexual dysfunction rate and de novo urinary dysfunction (stress and urgency-related incontinence) rate, intestinal impairment rate, pain after surgery. Finally, follow-up data were extracted, such as mesh-related complications (i.e., vaginal erosion of the mesh), de novo posterior POP diagnosis, relapse rate and need for re-operation rate due to POP. Length of follow-up was also collected.

### Quality assessment

Two independent reviewers (AL, CF) assessed bias risk in the included studies. Study quality was evaluated using the ROB2 tool [11] for randomized studies and the risk of Bias in non-randomized studies—of Interventions (ROBINS-I) scale [12] for observational studies.

### Data synthesis

A meta-analysis was conducted using the generic inverse-variance method with a random-effect model, provided sufficient data from at least three studies were available. The mean difference with 95% confidence intervals (CI) was computed for continuous variables, while the odds ratio (OR) with 95% two-sided confidence intervals (CI) was used for categorical variables. Heterogeneity among groups was assessed using the *I*-squared (*I*^2^) statistic, where an *I*^2^ value of less than 25% indicates low heterogeneity and an *I*^2^ value greater than 75% indicates high heterogeneity.

Meta-analysis was conducted using the Review Manager Web software, version 7.9.0 (The Nordic Cochrane Center, The Cochrane Collaboration, Copenhagen, Denmark); *p* value below 0.05 was considered statistically significant.

## Results

### Study selection

**Fig. 1:**
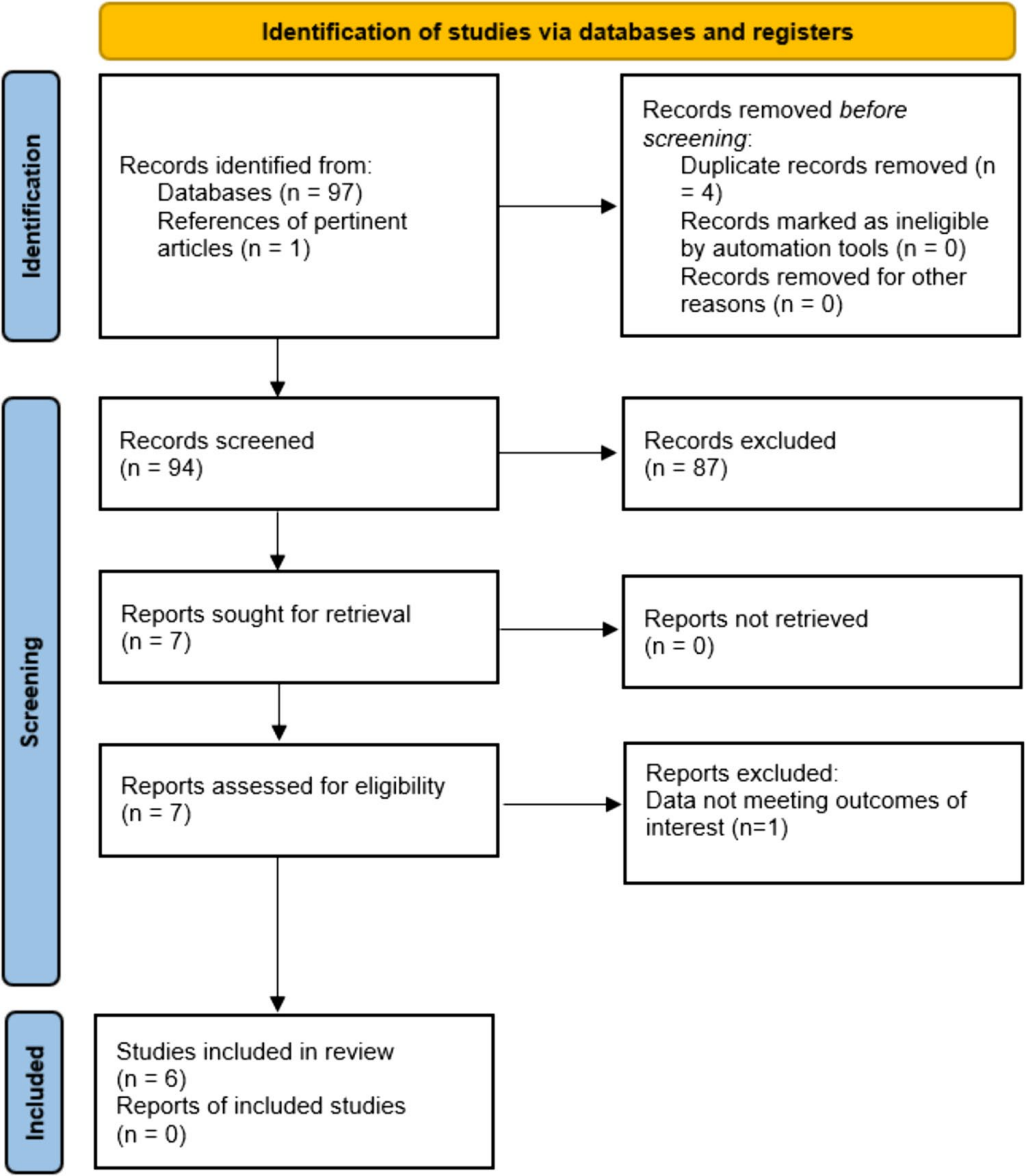
PRISMA flowchart of article selection phases

The search strategy uncovered a total of 97 articles. Once duplicates (4 articles) were eliminated, 93 unique articles were screened by reviewing titles and abstracts to identify those that met the inclusion criteria. Full-text copies of the remaining 6 articles were obtained and analyzed again for eligibility. Then, one article [13] was excluded as it did not provide data of interest according to the main objectives of this study. One article [14] was included by reviewing lists of references from pertinent articles. Figure 1 provides a detailed overview of the study inclusion process.

The final analysis comprised a total of six studies: three randomized controlled trials (RCT) [15–17] and three observational studies [14, 18, 19], including a total of 632 patients. Among included patients, 263 women had undergone laparoscopic sacral colpopexy (LSCP) with or without concurrent hysterectomy (either total or subtotal), whereas 369 women had undergone laparoscopic lateral suspension (LLS) with or without concurrent hysterectomy (either total or subtotal). For each study, data of interest was reported for LSCP (Group 1) and LLS (Group 2) in tables.

### Quality assessment

According to ROB2 tool and ROBINS-I scale, the risk of bias assessment showed an overall moderate risk of bias due to concerns about confounding variables (Figures S2 and S3). Among included RCTs, one [15] was concerned as high risk in terms of attrition and reporting bias; moreover, outcomes data regarding objective success were not unequivocally reported in tables and clearly interpretable; therefore, such data from that article were not extracted and incorporated in our meta-analysis (see Supplemental Materials-Appendix S2).

### Studies and baseline patient’s characteristics

Selected studies were published between 2022 and 2024. The inclusion criteria encompassed women diagnosed with Pelvic Organ Prolapse Stage Quantification (POP-Q) stage two or higher in apical prolapse, with or without anterior compartment prolapse. However, it should be noted that the study by Russo et al. [14] incorporated cases of multicompartmental prolapse in the LSCP group, while Tagliaferri et al. [19] also included POP-Q anterior compartment prolapse equal to or greater than stage one. All studies excluded women with posterior vaginal wall defects from their populations. Regarding the type of mesh used for suspension, most of included studies used a T shaped mesh for LLS, whereas different shapes of mesh were used for LSCP: V-shaped mesh [15], Y shaped mesh [14, 16], one strap mesh [17, 18] and double strap mesh [19]. Table S1 summarizes the general characteristics of included studies.

Regarding baseline patients’ characteristics, no significant differences about mean age (*P* = 0.27), parity (*P* = 0.36) and BMI (*P* = 0.62) were found among the groups being compared. None of included patients had undergone previous POP surgery (Table S2). Detailed data on the rate of advanced prolapse were reported in three out of six studies [14, 17, 18].

### Surgery-related data

**Fig. 2:**
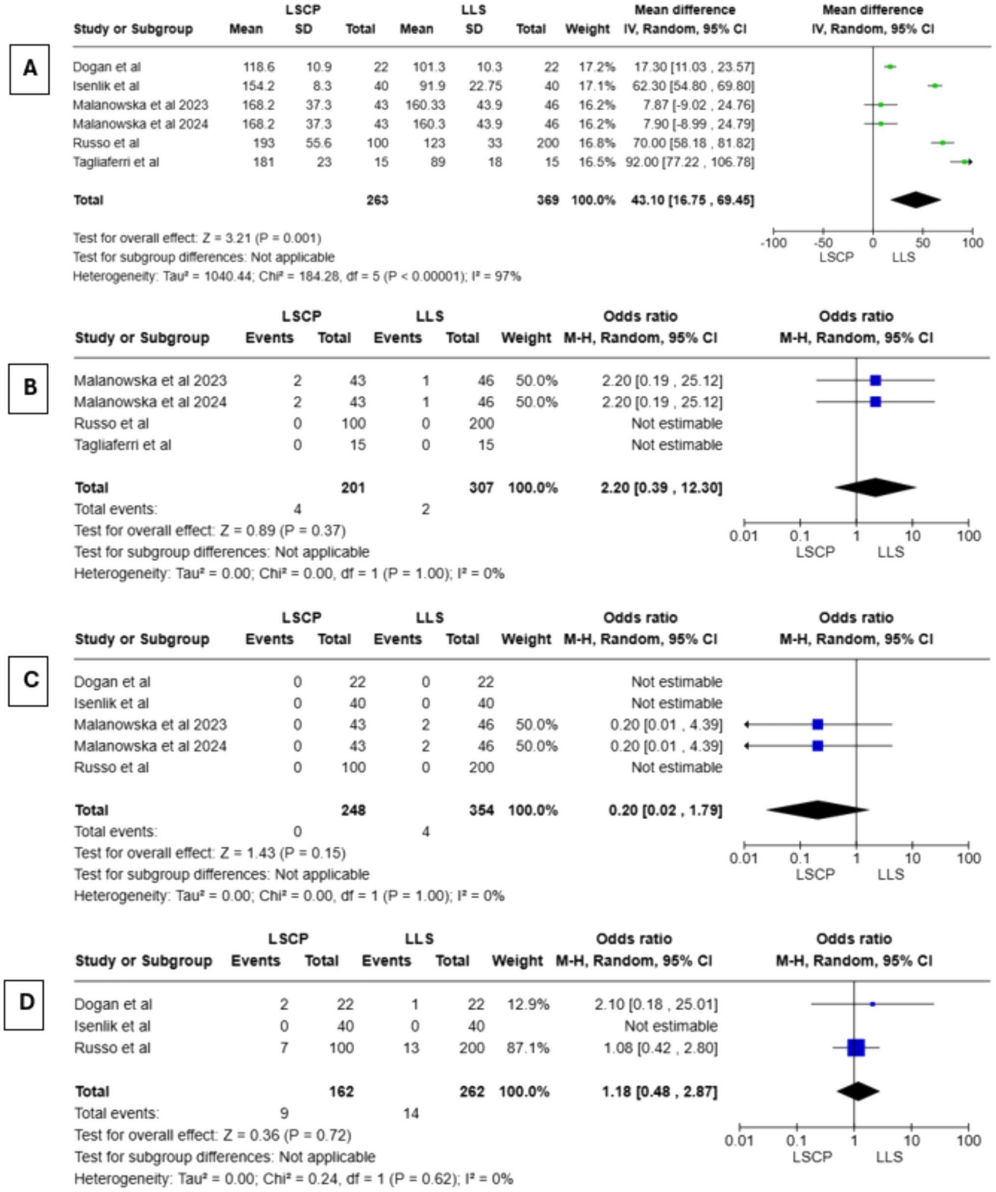
**a** Forest plot of operative time, **b** forest plot of laparotomy conversion rate, **c** forest plot of intraoperative complications, **d** forest plot of post-operative complications

Intraoperative and post-operative surgery-related data are shown in Tables S3 and S4. Regarding operative time, a statistically significant pool mean difference of 43.1 min was obtained in a random-effect meta-analysis [CI 95% (16.75, 69.45); *I*^2^ = 97%; *P* = 0.001] (Fig. 2A). However, analysis shows high heterogeneity (*I*^2^ = 97%), due to limited number of evidence, sensitivity analysis was not performed. This data takes into account whether a concomitant hysterectomy was performed or not (66.1% in LSCP group vs. 37.6% in LLS group). As shown in Fig. 2B–D, in our meta-analysis, using a random-effect model, no statistical differences were found regarding laparotomic conversion rate [OR = 2.20; CI 95% (0.39, 12.30); *I*^2^ = 0%; *P* = 0.37], intraoperative complications such as bladder, rectal and vaginal injuries [OR = 0.20; CI 95% (0.02, 1.79); *I*^2^ = 0%; *P* = 0.15] and early post-operative complications according to Clavien–Dindo classification [OR = 1.18; CI 95% (0.48, 2.87); *I*^2^ = 0%; *P* = 0.72]. Only two studies [16, 19] provided data about intraoperative blood loss, showing no significant differences among groups. Length of hospital stay was reported in two studies [14, 19] and was in most cases 2 days in both groups.

### Primary outcomes

**Fig. 3:**
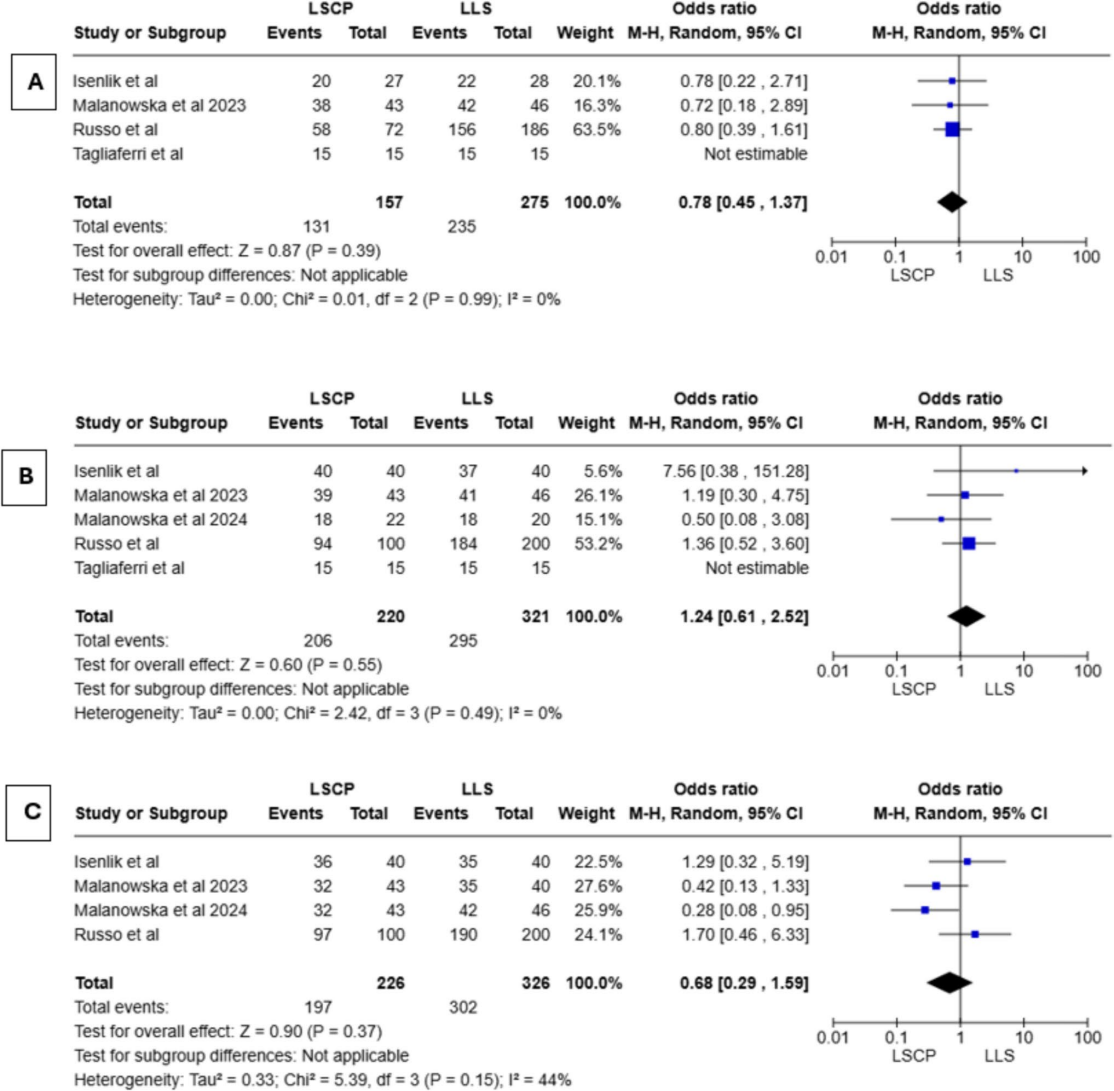
**a** Forest plot of objective success of anterior compartment, **b** forest plot of objective success of apical compartment, **c** forest plot of subjective success

As shown in Table S5, objective success was defined as a post-operative POP-Q stage lower than one [19], equal or lower than one [14, 15], lower than two [16, 18] and equal or lower than two [17] among different studies. Five studies out of six provided data about objective success according to anatomical compartments (i.e., apical and anterior); as discussed in the Quality Assessment paragraph it was not possible to extract such data from one study [15]. A random-effect meta-analysis shows no statistical differences between groups of comparison regarding the objective success of apical prolapse surgical correction [OR = 1.24; CI 95% (0.61, 2.52); *I*^2^ = 0%; *P* = 0.55] and anterior prolapse surgical correction [OR = 0.78; CI 95% (0.45, 1.37); *I*^2^ = 0%; *P* = 0.39]. Figure 3A, andB shows forest plot of objective success according to anatomical compartments. Objective success rate of apical and anterior compartments was 93.6 and 83.4% in the LSCP group and 91.9 and 85.4% in the LLS group, respectively.

Regarding subjective success, the included studies used different definitions, such as improvement in prolapse quality of life (P-QOL) score and pelvic organ prolapse–symptom score (POP–SS), absence of vaginal bulging and response “never” to the vaginal symptoms domain of the International Consultation on Incontinence Questionnaire–Vaginal Symptoms Module (ICIQ–VS) (Table S6). As reported in Fig. 3C subjective success was similar between groups of comparison [OR = 1.18; CI 95% (0.48, 2.87); *I*^2^ = 0%; *P* = 0.72].

### Follow-up data

Median length of follow-up was 14 months. Table S7 shows follow-up data strictly related to surgery. The majority of the included studies reported data about relapse of apical POP; therefore, a random-effect meta-analysis was conducted showing no statistical difference between LSCP and LLS [OR = 0.91; CI 95% (0.44, 1.88); *I*^2^ = 0%; *P* = 0.79]. Re-operation rate for POP correction was 3.4 and 4.2% in the LSCP and LLS group, respectively, without demonstrating a statistically significant difference between the groups [OR = 0.97; CI 95% (0.28, 3.43); *I*^2^ = 17%; *P* = 0.97]. Considering that posterior vaginal wall defects were an exclusion criteria in all included studies, three studies [16, 18, 19] provided data about de novo posterior POP. Using a random-effect meta-analysis no statistical difference emerged (*I*^2^ = 80%; *P* = 0.20). Sensitivity analysis was not performed due to paucity of data. Finally, mesh-related complications rate was 1.6% in LSCP group and 0.5% in LLS group, respectively (*P* = 0.24).

**Fig. 4:**
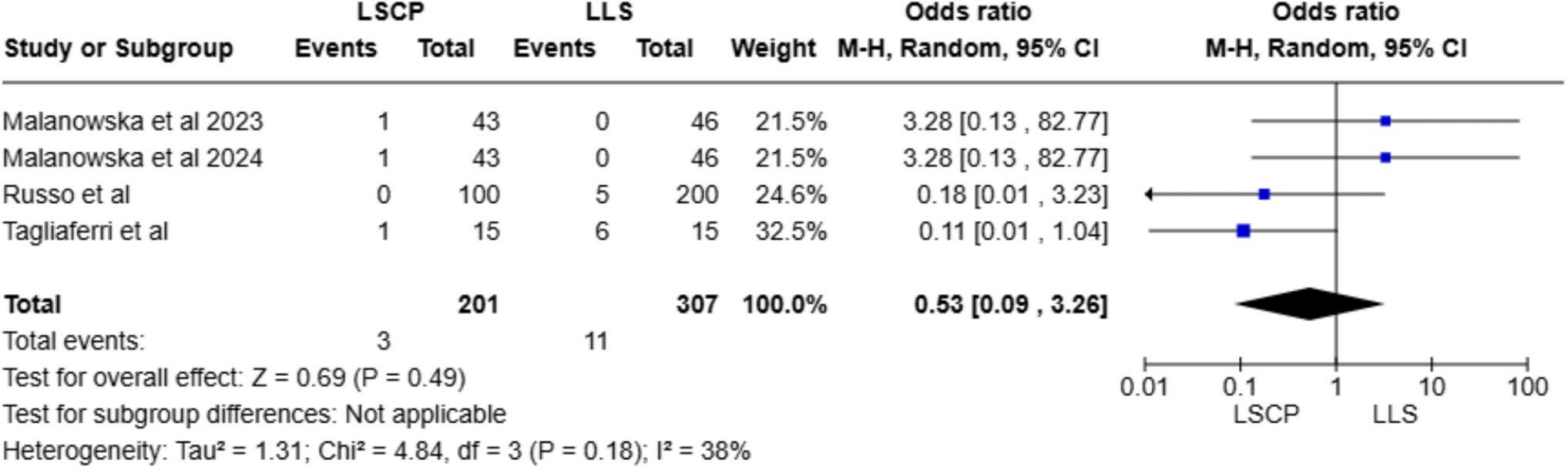
Forest plot of pain after surgery

Table S8 shows follow-up data connected with quality of life of patients. Regarding de novo urinary dysfunctions, i.e., stress urinary incontinence (SUI) and urge urinary incontinence (UUI), three studies [15, 16, 19] provided data on SUI leading to a meta-analysis. Statistical analysis shows no significant difference between comparison groups [OR = 0.91; CI 95% (0.21, 3.97); *I*^2^ = 15%; *P* = 0.90]. Intestinal impairment data such as constipation were provided by two studies [15, 19], showing slightly higher incidence of such symptom in the LLS group. Regarding sexual function, two studies [15, 16] reported an improvement in sexual activity from pre-operative to post-operative stages in both comparison groups. Finally, pain rates after surgery were assessed and extracted from four studies. In the LSCP group, pain was reported as back and lumbar pain, whereas in the LLS group, it was characterized as pain near the site of mesh fixation, near the anterior superior iliac spines. As shown in Fig. 4, the pain rate was slightly higher in the LLS group (3.5% in the LLS group vs. 1.4% in the LSCP group); however, the meta-analysis did not reach statistical significance [OR = 0.53; CI 95% (0.09, 3.26); *I*^2^ = 38%; *P* = 0.49].

## Conclusions

### Comparison with existing literature

According to recent data [4, 20], laparoscopic sacral colpopexy is considered the gold standard treatment for apical pelvic organ prolapse. Over the last decade, there has been increasing interest in less complex and less invasive surgical approaches. Currently, abdominal lateral suspension with mesh has emerged as a possible alternative for the treatment of apical POP, and a different abdominal mesh technique has been shown to be capable of achieving similar results in correcting both apical prolapse and anterior vaginal wall prolapse [8]. In our meta-analysis, LLS showed anatomical success rates comparable to LSCP while significantly reducing operative time. These results can be explained by the fact that this technique does not require deep pelvic dissection and is often associated with uterine preservation. In fact, a hysterectomy rate of 60% for sacral colpopexy compared to 30% for LLS has been demonstrated in the patients in this study. For this reason, there is insufficient evidence to definitively assess the impact of hysterectomy on operative time; future studies, including selected patients undergoing uterine-preservation techniques only, are strongly needed.

Despite the heterogeneity in its definition within the selected studies, the anatomical success rate of the anterior and apical compartments was similar between the two techniques. However, due to the short and medium-term follow-up periods reported, these results should be considered with caution, as they may not be sufficient to assess long-term results. Another key aspect in interpreting the results is the distribution of high-grade apical prolapse in the baseline population of the selected studies. Three [14, 17, 18] out of six studies provided some data on the prevalence of high-grade prolapse at baseline, and two [14, 17] of these reported a rate of advanced apical prolapse in approximately 50% of patients before surgery. Consequently, we cannot state that LLS can achieve comparable results to LSCP in relieving advanced apical prolapse, as selection bias cannot be fully excluded in our meta-analysis. Considering these aspects, comparative studies with longer follow-up and in patients with a higher risk of recurrence, such as those with stage three or higher apical prolapse, are needed to confirm the equivalency of the two techniques. Moreover, it should be noted that the population studied in these trials did not include patients with significant posterior vaginal prolapse, except one [14]. Although the rate of new onset of posterior prolapse is comparable between groups, it is important to consider that the presence of a posterior vaginal prolapse prior to surgery would likely expose the LLS group to higher rates of posterior recurrence. This can be explained by the LLS technique not involving dissection and reinforcement of the rectovaginal space [19]. In addition, the ventralization of the vagina due to mesh traction may create space for the development of a subsequent enterocele or rectocele [21, 22]. The LSCP also has an anteriorizing effect on the axis of the vagina [23]; yet, according to recent literature, it should be emphasized that only LCSP can be associated with integrated treatment of the posterior compartment and rectal prolapse [24, 25]. Reliable studies investigating the combination of LLS with posterior compartment reconstructive techniques are currently limited. At present, it is not possible to consider these techniques as direct alternatives, but they should be considered as therapeutic options specifically suited for a group of patients presenting apical prolapse in the absence of posterior defects.

The recurrence rates of apical prolapse are comparable between the two techniques (6% vs. 7%), consistently with the success rate reported for abdominal prosthetic procedures [4]. It should be noted that the included studies do not provide details on managing recurrent apical prolapse, with the exception of Isenlik et al.[16] series, where LLS relapses were treated with subsequent LSCP. In this regard, the literature offers insufficient evidence to consider LLS as a suitable option, while LSCP remains the treatment of choice for recurrent POP [26].

Mesh-related complications occurrence was similar between groups, showing an incidence rate lower than 2%, which is consistent with data in literature [27, 28]. Mesh erosion after LSCP is statistically associated with concomitant total hysterectomy [29, 30]. Regarding LLS, which is generally a uterine-preservation technique, some authors [31] suggest that mesh erosion is mainly related to the use of macroporous meshes with either multifilamentous or microporous components and to the use of non-absorbable suture to fix the mesh to the vesico-vaginal fascia.

Regarding follow-up data, this study does not exclusively support the benefits of POP surgery from an anatomical point of view. We reported an overall improvement in sexual dysfunction and a low rate of post-operative functional impairment, such as urinary and intestinal dysfunctions. This confirms the efficacy of both techniques in improving quality of life recovery. Post-operative pain after POP surgery is still poorly investigated in literature and often under-estimated in clinical practice. According to extracted data, LSCP seems to be more frequently associated with back and lumbar pain, which can be explained by sacral suspension of the mesh. In this regard, it is of utmost importance to recognize “alarming” symptoms of spondylodiscitis, a rare mesh-related complication of LSCP [32, 33]. Instead, LLS exhibits more frequently myofascial pain syndrome showing sites of mesh fixation (i.e., area near the anterior superior iliac spines) as potential myofascial trigger points. Currently, there is insufficient data to speculate on long-term outcomes, especially in young patients who lead active lives. At present date, a recent Delphi survey of expert panel [34] stated that fixation of the prosthesis to the abdominal muscle fascia is no longer necessary and should be avoided, as it is a procedure linked to post-operative pain. No data are currently available on mesh tension and post-operative pain level, which require additional investigation in further studies.

### Strengths and limitations

To our knowledge, this is the first meta-analysis comparing LLS and LSCP. We analyzed a significant amount of data, including objective and mid-term follow-up findings, to support the development of evidence-based guidelines. Moreover, the data exhibited mainly low to moderate heterogeneity (*I*^2^). Despite these strengths, a major limitation is the small number of studies that met the inclusion criteria. In addition, the differences in outcome reporting among the included studies may introduce variability and potential bias into the analysis. The overall risk of bias in the analyzed studies ranged from moderate to high, underscoring the need for cautious interpretation of the results.

### Conclusions

This study demonstrated comparable results in terms of anatomical success rate in treating anterior and apical prolapse between LSCP and LLS in selected patients with short-to-medium-term follow-up. Operative time is shorter with LLS, but the rate of hysterectomy has been reported to be twice as high in the LSCP population. Re-intervention, complication, and recurrence rates were similar between the two approaches. The lack of long-term follow-up and the paucity of data on patients with advanced apical prolapse do not allow LLS to be declared an alternative treatment with equal effects to LSCP. There is a clear need for further research using larger cohorts, longer follow-up periods, and standardized methods for reporting outcomes to confirm and expand upon the findings of this study.

## Supplementary Information

Below is the link to the electronic supplementary material.

Supplementary file1 (DOCX 861 KB)

## Data Availability

All data relevant to the study are included in the article or uploaded as supplementary information. All data were extracted from previously published studies; thus, they are publicly available.
