# Supplementary material for: Lateral suspension vs. sacral colpopexy for treating pelvic organ prolapse: a systematic review and meta-analysis
Source: Arch Gynecol Obstet. 2025 Oct 3;312(6):1891–900. doi: 10.1007/s00404-025-08210-4 (PMC12705715; doi:10.1007/s00404-025-08210-4)

**Supporting Information**

**Lateral suspension versus sacral colpopexy for treating pelvic organ prolapse: a systematic review and meta-analysis**

Andrea Lombisani^1#^, Veronica Tius^2#^, Chiara Ferraro^3^, Martina Arcieri^4^, Lorenzo Vacca^1^, Daniela Caramazza^1^, Stefano Restaino^4,5^, Tommaso Simoncini^6^, Giampiero Capobianco^7^, Alfredo Ercoli^8^, Giovanni Scambia^3,9^, Giuseppe Vizzielli^2,4†^, Giuseppe Campagna^1†^

^#^contributed equally

^†^contributed equally

*Correspondence to: Prof. Giuseppe Vizzielli - Medical Area Department (DAME), in Department of Medicine (DMED), University of Udine, piazzale S. Maria della Misericordia 15 - Udine, Italy. Email: [giuseppevizzielli@yahoo.it](mailto:giuseppevizzielli@yahoo.it); giuseppe.vizzielli@uniud.it. Tel: 0432559695

**Appendix S1: Search Strategy**

Pubmed:

(((((pelvic organ prolapse) OR (urogenital prolapse)) AND (lateral suspension))

Google Scholar:

Search phrase “Comparison between sacral colpopexy and lateral suspension for pelvic organ prolapse”

**Appendix S2: Risk of bias for individual studies with support for judgment**

Risk of Bias – Dogan (RCT)

| Bias | Author’s judgement | Support for judgement |
| --- | --- | --- |
| Random sequence generation: SELECTION BIAS | Low risk | The randomization process followed a permuted block design with block size 2, maintaining an equal 1:1 allocation ratio between the groups. |
| Allocation concealment: SELECTION BIAS | Intermediate risk | An independent team managed the random assignment to the respective groups but there is no statement that allocation was concealed or whether the team was blinded |
| Blinding of participants and personnel: PERFORMANCE BIAS | Low risk | The care provider remained blinded to the type of operation. |
| Blinding of outcome assessment: DETECTION BIAS | Low risk | The assessment of outcomes was conducted by a gynecologist who was also unaware of the operation type, indications, and complications. |
| Incomplete outcome data: ATTRITION DATA | High risk | The analysis included 44 eligible patients (lateral suspension, 22; sacrouteropexy, 22). No details are provided on patient dropout or loss to follow-up; the pandemic-related delay may have led to participant dropout or irregular follow-up, but no information is given on whether all randomized patients completed the study and no flow diagram is reported. Although the final sample size equals the number randomized, the absence of these details justify a high risk of attrition bias. |
| Selective reporting: REPORTING BIAS | High risk | Adherence to CONSORT requires disclosure of prespecified primary and secondary outcomes and also registration details, which are not clearly provided. Outcomes like reoperation and recurrence are only mentioned but not systematically analyzed or discussed |
| Other bias |  |  |

Risk of Bias – Isenlik (RCT)

| Bias | Author’s judgement | Support for judgement |
| --- | --- | --- |
| Random sequence generation: SELECTION BIAS | Low risk | Randomization to groups was performed by an independent secretariat working at the hospital employing block randomization (permuted block size of 2 with a 1:1 allocation ratio). |
| Allocation concealment: SELECTION BIAS | Low risk | The group allocations were concealed in opaque (sealed) envelopes. |
| Blinding of participants and personnel: PERFORMANCE BIAS | High risk | Neither patients nor surgeons were blinded, introducing high risk of performance bias. |
| Blinding of outcome assessment: DETECTION BIAS | Moderate risk | Evaluations of the primary and secondary outcomes were performed at baseline and 12 months post-surgery by a physician blinded to the intervention. The subjective nature of some secondary outcomes and lack of further blinding details justify a moderate risk. |
| Incomplete outcome data: ATTRITION DATA | Low risk | The flowchart shows no lost follow-up; all 80 randomized patients were followed and analyzed. |
| Selective reporting: REPORTING BIAS | Low risk | The trial was pre-registered, and all outcomes (primary and secondary) described in the methods are reported in the results, indicating low risk of selective reporting. |
| Other bias |  |  |

Risk of Bias – Malanowska 2024 (RCT)

| Bias | Author’s judgement | Support for judgement |
| --- | --- | --- |
| Random sequence generation: SELECTION BIAS | Low risk | Participants were randomly assigned (1:1) to either the laparoscopic lateral suspension (LLS) group or the laparoscopic sacropexy (LS) group. |
| Allocation concealment: SELECTION BIAS | Low risk | The allocation sequence was created using a sealed envelope system, ensuring that the allocation was concealed. |
| Blinding of participants and personnel: PERFORMANCE BIAS | High risk | At follow-up, the medical doctor was blinded to the group intervention allocation. However, there is no mention of blinding the participants or the personnel performing the interventions. This lack of blinding for participants and personnel suggests a high risk of performance bias. |
| Blinding of outcome assessment: DETECTION BIAS | Moderate risk | At follow-up, the medical doctor was blinded to the group intervention allocation. The subjective nature of some outcomes and lack of further blinding details justify a moderate risk. |
| Incomplete outcome data: ATTRITION DATA | Low risk | A total of 93 women were randomized, with 2 women lost to follow-up in both the LLS and LSC groups. Four patients were lost to follow-up in total, with 89 women completing the 1-year follow-up. This indicates a low rate of incomplete outcome data. |
| Selective reporting: REPORTING BIAS | Moderate risk | The results section presents data on anatomical and functional outcomes, operative time, complications and patient datisfaction. Without access to the study protocol or registration, it is difficult to definitively confirm that all pre-specified outcomes were reported, leading to a moderate risk assessment. |
| Other bias |  |  |

Risk of Bias – Malanowska 2023 (observational)

| Bias | Author’s judgement | Support for judgement |
| --- | --- | --- |
| Bias due to confounding | Intermediate risk | All patients were evaluated preoperatively with a physical examination, assessing uterine prolapse using the POP-Q system, and a standardized PFDI-20 questionnaire. While baseline characteristics were assessed, the study does not detail how potential confounding factors beyond these baseline measures were handled in the analysis leading to an intermediate risk assessment. |
| Bias in selection of participants into the study | High risk | The specific recruitment process and potential for selection bias during enrollment are not fully detailed. |
| Bias in classification of interventions | Intermediate risk | The details regarding the standardization of the surgical procedures or the training/experience of the surgeons performing them are not extensively described, which could lead to some variability in how the interventions were classified or performed in practice. This warrants an intermediate risk. |
| Bias due to deviations from intended interventions | Intermediate risk | There is no information provided about monitoring adherence to the surgical protocols, or if any participants deviated from their assigned intervention. |
| Bias due to missing data | Intermediate risk | 4 patients lost the follow-up. While the number of missing participants is small, the method used to handle this missing data in the analysis is not explicitly detailed. |
| Bias in measurements of outcomes | Low risk | The outcomes were measured using objective methods. Furthermore, the medical doctor was blinded to the group intervention allocation, which reduces the potential for bias in outcome assessment. |
| Bias in selection of the reported result | Intermediate risk | Without access to the study protocol, it's challenging to confirm that all pre-specified outcomes were reported and that no selective reporting occurred. |
| Overall bias |  |  |

Risk of Bias – Russo (observational)

| Bias | Author’s judgement | Support for judgement |
| --- | --- | --- |
| Bias due to confounding | Moderate risk | The text does not explicitly detail how potential confounding factors beyond baseline characteristics were handled in the analysis, which could influence the outcomes. |
| Bias in selection of participants into the study | Moderate risk | The detailed process of how participants were selected across multiple centers is not fully elaborated. |
| Bias in classification of interventions | Low Risk | The nature of a multicenter trial typically aims for uniformity, leading to a low risk assumption for classification. |
| Bias due to deviations from intended interventions | Moderate risk | There is no explicit information provided about how adherence to the assigned interventions was monitored or if there were any significant deviations from the intended procedures among participants. |
| Bias due to missing data | Low risk | No specific details regarding patient follow-up rates or the handling of missing data are shown. However, it is stated that women who were reluctant to return for follow-up were previously excluded  from this study. |
| Bias in measurements of outcomes | Moderate risk | The text does not specify if outcome assessors were blinded to the intervention groups. |
| Bias in selection of the reported result | Low risk | The study is likely to have pre-specified primary and secondary outcomes, and the pre-defined outcomes are consistently reported. |
| Overall bias | Moderate risk |  |

Risk of Bias – Tagliaferri (observational)

| Bias | Author’s judgement | Support for judgement |
| --- | --- | --- |
| Bias due to confounding | Moderate risk | In retrospective studies, it's challenging to control for all potential confounding factors that might have influenced treatment assignment or outcomes. |
| Bias in selection of participants into the study | High risk | No detailed inclusion/exclusion criteria or the method of participant selection from the overall patient population are specified, which could lead to a non-random selection of participants. Retrospective studies often result in a high risk of selection bias, as the choice of intervention was not random and may have been influenced by factors that also affect the outcome. |
| Bias in classification of interventions | Moderate risk | In a retrospective setting, there might be variations in how these procedures were performed over time or by different surgeons. The text doesn't provide details on the standardization of these interventions or surgeon experience, which could lead to some heterogeneity. |
| Bias due to deviations from intended interventions | Low risk | Since the study is analyzing procedures that have already been performed, it's assumed that the patients received the intended intervention for which they were classified. |
| Bias due to missing data | Moderate risk | The study is retrospective, relying on existing medical records. It is not mentioned how complete the data was for all patients, nor is it specified the percentage of missing data. In retrospective studies, incomplete documentation is common and it can bias the results. |
| Bias in measurements of outcomes | Moderate risk | In a retrospective study, outcomes are usually extracted from patient records and the original clinicians or data extractors may not have been blinded. Moreover, it is stated that post-operative follow-up was performed by the same surgical equipe.  This could introduce bias in outcome measurements. |
| Bias in selection of the reported result | Moderate risk | As a retrospective study, there is a risk that the selection of outcomes reported might be influenced by the observed results, leading to selective reporting bias. Without a pre-registered protocol about all outcomes, it's difficult to say if all relevant findings were reported or if there was an emphasis on statistically significant outcomes. |
| Overall bias | Moderate risk |  |

**Table S1. Characteristics of included studies.**

| Study | Year | Country | Type of study | Type of Prolapse (inclusion criteria) | Type of Prolapse (Exclusion criteria) | Group of comparison | Type of mesh | Number of patients | |
| --- | --- | --- | --- | --- | --- | --- | --- | --- | --- |
|  |  |  |  |  |  |  |  | Group 1 | Group 2 |
| Dogan et al | 2024 | Turkey | RCT | POP-Q apical prolapse stage ≥ 2  with or without anterior compartment prolapse | NA | LSCP vs LLS +/- McCall Culdoplasty | V- shaped mesh | 22 | 22 |
| Isenlik et al | 2023 | Turkey | RCT | POP-Q apical prolapse stage ≥ 2  with or without anterior compartment prolapse | posterior vaginal wall defects | LSCP-TH vs LLS-TH | Y-shaped mesh for LSCP / T- shaped mesh for LLS | 40 | 40 |
| Malanowska et al | 2023 | Poland-Italy | OBS | POP-Q apical prolapse stage ≥ 2  with or without anterior compartment prolapse | posterior vaginal wall defects | LSCP + STH +/- SO vs LLS + STH +/- SO | One strap mesh for LSCP / T-shaped mesh for LLS | 43 | 46 |
| Malanowska et al | 2024 | Poland-Italy | RCT | POP-Q apical prolapse stage ≥ 2  with or without anterior compartment prolapse | posterior vaginal wall defects | LSCP + STH +/- SO vs LLS + STH +/- SO | One strap mesh for LSCP / T-shaped mesh for LLS | 43 | 46 |
| Russo et al | 2023 | Italy | OBS | LSCP: multicompartimental POP-Q stage ≥ 2; LLS: anterior and apical prolapse POP-Q stage ≥ 2 | advanced posterior vaginal defect for LLS | LSCP +/- STH vs LLS +/- STH | Y-shaped mesh for LSCP / T-shaped mesh for LLS | 100 | 200 |
| Tagliaferri et al | 2022 | Italy | OBS | POP-Q apical stage ≥ 2, POP-Q anterior compartment ≥ 1 | posterior vaginal wall defects | LCSP + STH vs LLS +/- TH | Double mesh for LSCP / T-shaped mesh for LLS | 15 | 15 |

RCT: Randomized Controlled Trial; OBS: observational study; POP-Q: Pelvic Organ Prolapse- Quantification System; LSCP: laparoscopic sacral colpopexy; LLS: laparoscopic lateral suspension; TH: total hysterectomy; STH: subtotal hysterectomy; SO: salpingo-oophorectomy;

**Table S2. Baseline patients’ characteristics.**

| Study | Age (years) | | Parity | | BMI (kg/m^2^) | | Previous POP surgery | | Advanced POP stage (III-IV) | |
| --- | --- | --- | --- | --- | --- | --- | --- | --- | --- | --- |
|  | Group 1 | Group 2 | Group 1 | Group 2 | Group 1 | Group 2 | Group 1 | Group 2 | Group 1 | Group 2 |
| Dogan et al  2024 | 52,8 ± 8,6 | 51,2 ± 8,4 | 2,5 ± 2 | 3 ± 2,2 | 28,5 ± 3,6 | 28,8 ± 2,9 | 0/22 (0%) | 0/22 (0%) | ^a^ | ^a^ |
| Isenlik et al  2023 | 59,4 ± 8,8 | 59,4 ± 8,6 | 2 (0-8) | 3 (1-6) | 27,3 (19,1-37,8) | 27,5 (20-35,8) | 0/40 (0%) | 0/40 (0%) | NA | NA |
| Malanowska et al  2023 | NA | NA | NA | NA | NA | NA | 0/43 (0%) | 0/46 (0%) | 22 (51,2%) | 20 (43,48%) |
| Malanowska et al  2024 | 58,1 ± 8,2 | 59,4 ± 8,8 | 2,2 ± 1,03 | 2,4 ± 1,1 | 26,5 ± 3,7 | 25,9 ± 3,7 | 0/43 (0%) | 0/46 (0%) | APEX: 22/43 (51,1%)  ANTE: 16/43 (37,2%) | APEX: 20/46 (43,4%)  ANTE: 17/46 (36,9%) |
| Russo et al  2023 | 64,9 ± 8,8 | 63,1 ± 8,1 | NA | NA | 24,5 ± 1,8 | 24 ± 2,4 | NA | NA | APEX: 85/100 (85%) ANTE: 57/100 (57%) POST: 31/100 (31%) | APEX: 146/200 (73%) ANTE: 157/200 (78,5%) POST: 2/200 (1%) |
| Tagliaferri et al  2022 | 57 ± 7 | 57 ± 7 | 2,5 ± 0,5 | 2,5 ± 1,1 | 25,3 ± 2,6 | 26,8 ± 3 | 0/15 (0%) | 0/15 (0%) | NA | NA |

^a^ Data was not extracted due to not interpretable tables

BMI: Body Mass Index; POP: pelvic organ prolapse; NA: not avalaible; APEX: apical compartment; ANTE: anterior compartment; POST: posterior compartment

**Table S3. Surgery-related data.**

| Study | Operative time (min) | | Concomitant hysterectomy | | LPT Conversion rate | |
| --- | --- | --- | --- | --- | --- | --- |
|  | Group 1 | Group 2 | Group 1 | Group 2 | Group 1 | Group 2 |
| Dogan et al  2024 | 118,6 ± 10,9 | 101,3 ± 10,3 | 0/22 (0%) | 0/22 (0%) | NA | NA |
| Isenlik et al  2023 | 154,2 ± 8,3 | 91,95 ± 22,75 | 40/40 (100%) | 40/40 (100%) | NA | NA |
| Malanowska et al  2023 | 168,26 ± 37,37 | 160,33 ± 43,91 | 43/43 (100%) | 46/46 (100%) | 2/43 (4,7%) | 1/46 (2,1%) |
| Malanowska et al  2024 | 168,26 ± 37,37 | 160,33 ± 43,91 | 43/43 (100%) | 46/46 (100%) | 2/43 (4,6%) | 1/46 (2,1%) |
| Russo et al  2023 | 193 ± 55,6 | 123 ± 33 | 33/100 (33%) | 6/200 (3%) | 0/100 (0%) | 0/200 (0%) |
| Tagliaferri et al  2022 | 181 ± 23 | 89 ± 18 | 15/15 (100%) | 1/15 (6,6%) | 0/15 (0%) | 0/15 (0%) |

LPT: laparotomy; NA: not available

**Table S4. Surgery-related data.**

| Study | Intraoperative Complications (bladder, rectal, vaginal injury) | | Early post operative complications (Clavien Dindo classification) | | Blood Loss (ml) | | Hospital Stay (day) | |
| --- | --- | --- | --- | --- | --- | --- | --- | --- |
|  | Group 1 | Group 2 | Group 1 | Group 2 | Group 1 | Group 2 | Group 1 | Group 2 |
| Dogan et al  2024 | 0/22 (0%) | 0/22 (0%) | 2/22 (9,1 %) | 1/22 (4,5 %) | NA | NA | NA | NA |
| Isenlik et al  2023 | 0/40 (0%) | 0/40 (0%) | 0/40 (0%) | 0/40 (0%) | 104,75 ± 62,39 | 94,38 ± 59,05 | NA | NA |
| Malanowska et al  2023 | 0/43 (0%) | 2/46 (4,3%) | NA | NA | 100 | 90 | NA | NA |
| Malanowska et al  2024 | 0/43 (0%) | 2/46 (4,3%) bladder | NA | NA | NA | NA | NA | NA |
| Russo et al  2023 | 0/100 (0%) | 0/200 (0%) | 7/100 (7%) | 13/200 (6,5%) | NA | NA | 2 | 2 |
| Tagliaferri et al  2022 | NA | NA | NA | NA | 62 ± 39 | 64 ± 21 | 2,2 ± 0,4 | 2 ± 0,7 |

NA: not available

**Table S5. Objective outcomes.**

| Study | Objective success | | |
| --- | --- | --- | --- |
|  | Definition | Group 1 | Group 2 |
| Dogan et al  2024 | POP-Q stage ≤ 1 | ^a^ | ^a^ |
| Isenlik et al  2023 | POP-Q stage < 2 | APEX: 40/40 (100%) ANTE: 20/27 (74,1%) | APEX: 37/40 (92,5%) ANTE: 22/28 (78,6 %) |
| Malanowska et al  2023 | POP-Q stage < 2 | APEX: 39/43 (90,7%) ANTE: 38/43 (88,37%) | APEX: 41/46 (89,1%) ANTE: 42/46 (91,3%) |
| Malanowska et al  2024 | POP-Q stage ≤ 2 | APEX: 18/22 (81,2%) ANTE + APEX: 20/21 (95,22%) TOTAL: 38/43 (88,37%) | APEX: 18/20 (92,3%) ANTE + APEX: 24/26 (92,3%) TOTAL: 42/46 (91,3%) |
| Russo et al  2023 | POP-Q stage ≤ 1 | APEX: 94/100 (94%) ANTE: 58/72 (80,6%) POST: 47/55 (85,4%) | APEX: 184/200 (92%) ANTE: 156/186 (83,9%) POST: 6/12 (50%) |
| Tagliaferri et al  2022 | POP-Q stage < 1 | APEX: 15/15 (100%) ANTE:15/15 (100% | APEX: 15/15 (100%) ANTE:15/15 (100% |

^a^Data was not extracted due to not interpretable tables

POP-Q: Pelvic Organ Prolapse- Quantification System; APEX: apical compartment; ANTE: anterior compartment; POST: posterior compartment

**Table S6. Subjective outcome**

| Study | Subjective success | | |
| --- | --- | --- | --- |
|  | Definition | Group 1 | Group 2 |
| Dogan et al  2024 | Improvement in PQOL and POP-SS score | PRE POP-SS total score: 20,18 ± 5,27 POST POP-SS total score: 5,9 ± 3,62 | PRE POP-SS total score: 21,22 ± 5,4 POST POP-SS total score: 6,22 ± 3,45 |
| Isenlik et al  2023 | response "never" in the vaginal symptoms domain of the ICIQ-VS questionnaire | 36/40 (90%) | 35/40 ( 87,5%) |
| Malanowska et al  2023 | absence of bulging (PFDI-20 questionnaire) | 32/43 (74,4%) | 42/46 (91,3%) |
| Malanowska et al  2024 | absence of bulging (PFDI-20 questionnaire) | 32/43 (74,4%) PRE PFDI-20 score: 106,81 POST PFDI-20 score: 20,11 | 42/46 (91,3%) PRE PFDI-20 score: 102,02 POST PFDI-20 score: 16,24 |
| Russo et al  2023 | absence of vaginal bulge | 97/100 (97%) | 190/200 (95%) |
| Tagliaferri et al  2022 | Improvement in PQOL score | NA | NA |

P-QOL:Prolapse Quality of Life; POP-SS: Pelvic Organ Prolapse- Symptom Score; ICIQ-VS: International Consultation on Incontinence Questionnaire Vaginal Symptoms Module; PFDI-20: Pelvic Floor Disability Index; PRE: pre-operative; POST: post-operative

**Table S7. Follow up data.**

| Study | Mesh related complications | | Relapse | | Need for reoperation for POP | | De novo posterior POP | | Lenght of follow up (months) |
| --- | --- | --- | --- | --- | --- | --- | --- | --- | --- |
|  | Group 1 | Group 2 | Group 1 | Group 2 | Group 1 | Group 2 | Group 1 | Group 2 |  |
| Dogan et al  2024 | NA | NA | NA | NA | 1/22 (4,5%) | 0/22 (0%) | NA | NA | 12 |
| Isenlik et al  2023 | 1/40 (2,5%) | 0/40 (0%) | APEX: 0/40 (0%) ANTE: 7/27 (25,9%) | APEX: 3/40 (7,5%) ANTE: 6/28 (21,4%) | 0/40 (0%) | 3/40 (7,5%) | 3/40 (7,5%) | 4/40 (10%) | 12 |
| Malanowska et al  2023 | 0/43 (0%) | 0/46 (0%) | APEX: 4/43 (9,3%) | APEX: 3/46 (6,5%) | 0/43 (0%) | 0/46 (0%) | 1/43 (2,3%) | 2/46 (4,3%) | 12 |
| Malanowska et al  2024 | 0/43 (0%) | 0/43 (0%) | APEX: 4/22 (18,1%) | APEX: 2/20 (10%) | NA | NA | NA | NA | 12 |
| Russo et al  2023 | 2/100 (2%) | 2/200 (1%) | APEX: 6/100 (6%) ANTE: 14/72 (19,4%) POST: 8/55 (14,6%) | APEX: 16/200 (8%) ANTE: 30/186 (16,1%) POST: 6/12 (50%) | 6/100 (6%) | 10/200 (5%) | NA | NA | 12 |
| Tagliaferri et al  2022 | 1/15 (6,6%) | 0/15 (0%) | 0/15 (0%) | 0/15 (0%) | NA | NA | 0/15 (0%) | 15/15 (100%) | 24 |

POP: pelvic organ prolapse: APEX: apical compartment; ANTE: anterior compartment; POST: posterior compartment; NA: nota available

**Table S8. Follow up data.**

| Study | De novo urinary dysfunction (SUI, UUI) | | Intestinal impairment (constipation) | | | Sexual dysfunction (dyspareunia, sexual discomfort) | | | Pain after surgery | |
| --- | --- | --- | --- | --- | --- | --- | --- | --- | --- | --- |
|  | Group 1 | Group 2 | Definition | Group 1 | Group 2 | Definition | Group 1 | Group 2 | Group 1 | Group 2 |
| Dogan et al  2024 | de novo SUI: 1/22 (4,5%) de novo UUI: 2/22 (9,1%) | de novo SUI: 0/22 (0%) de novo UUI: 3/22 (13,6%) | de novo constipation | 1/22 (4,5%) | 2/22 (9,1%) | Improvement in FSFI score | PRE: 15,8 ±4,42 POST: 23,69 ±3,66 | PRE: 15,45 ± 5,9 POST: 23,44 ± 3,9 | NA | NA |
| Isenlik et al  2023 | de novo SUI: 1/40 (2,5%) de novo UUI: 0/40 (0%) | de novo SUI: 4/40 (10%) de novo UUI: 2/40 (5%) | NA | NA | NA | SMS | PRE: 9,8 ± 14,35 POST: 3,15 ± 6,17 | PRE: 15,35 ± 16,41 POST: 2,35 ± 5,5 | NA | NA |
| Malanowska et al  2023 | NA | NA | NA | NA | NA | NA | NA | NA | 1/43 (2,3%) severe back pain | 0/46 (0%) |
| Malanowska et al  2024 | NA | NA | NA | NA | NA | NA | NA | NA | 1/43 (2,3%) severe back pain | 0/46 (0%) |
| Russo et al  2023 | NA | NA | NA | NA | NA | NA | NA | NA | 0/100 (0%) | 5/200 (2,5%) pain near the anterior superiori iliac spine |
| Tagliaferri et al  2022 | de novo SUI: 3/15 (20%) | de novo SUI: 2/15 (13,3%) | severe bowel symptoms | 0/15 (0%) | 7/15 (43,4%) | NA | NA | NA | 1/15 (6,6%) lumbar pain | 6/15 (40%) pain in the site of mesh fixation |

SUI: stress urinary incontinence; UUI: urge urinary incontinence; FSFI: FSFI: Female Sexual Function Index; SMS: Sexual Matter Score; PRE: pre-operative; POST: post-operative**;** NA: not available

**Fig. S2 ROB1 for randomized controlled trials summary and graph**


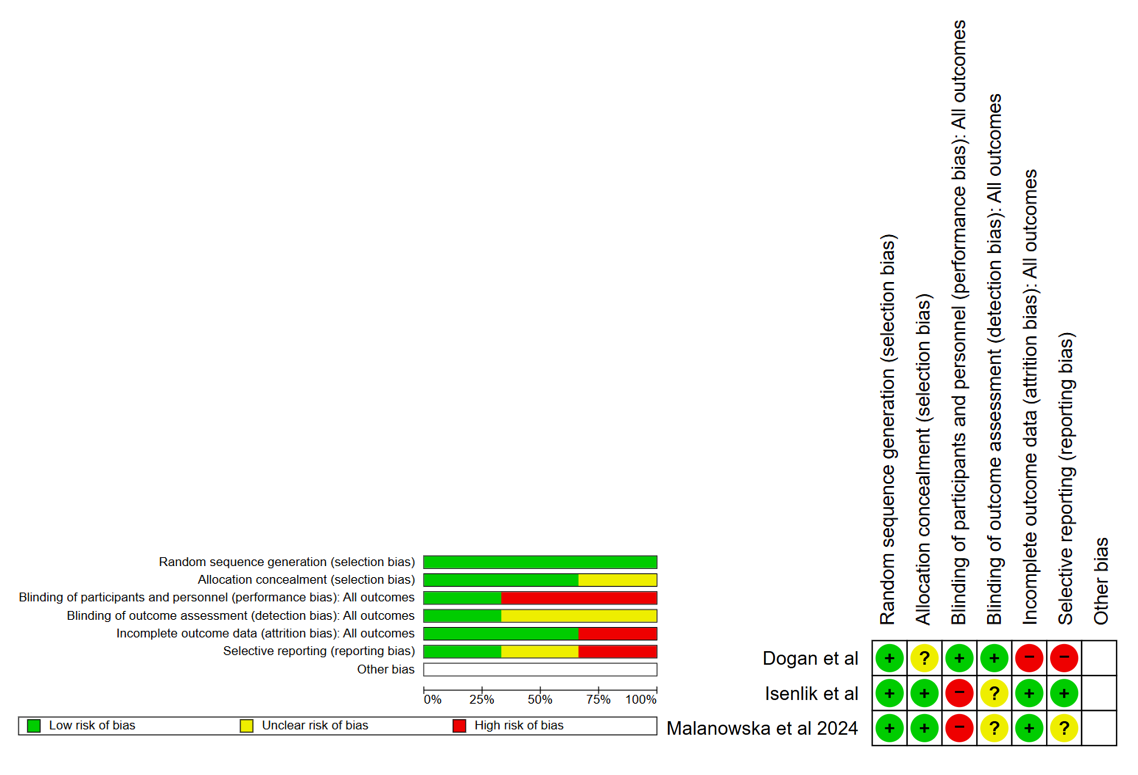


**Fig. S3 ROBINS-1 for observational study summary and graph**


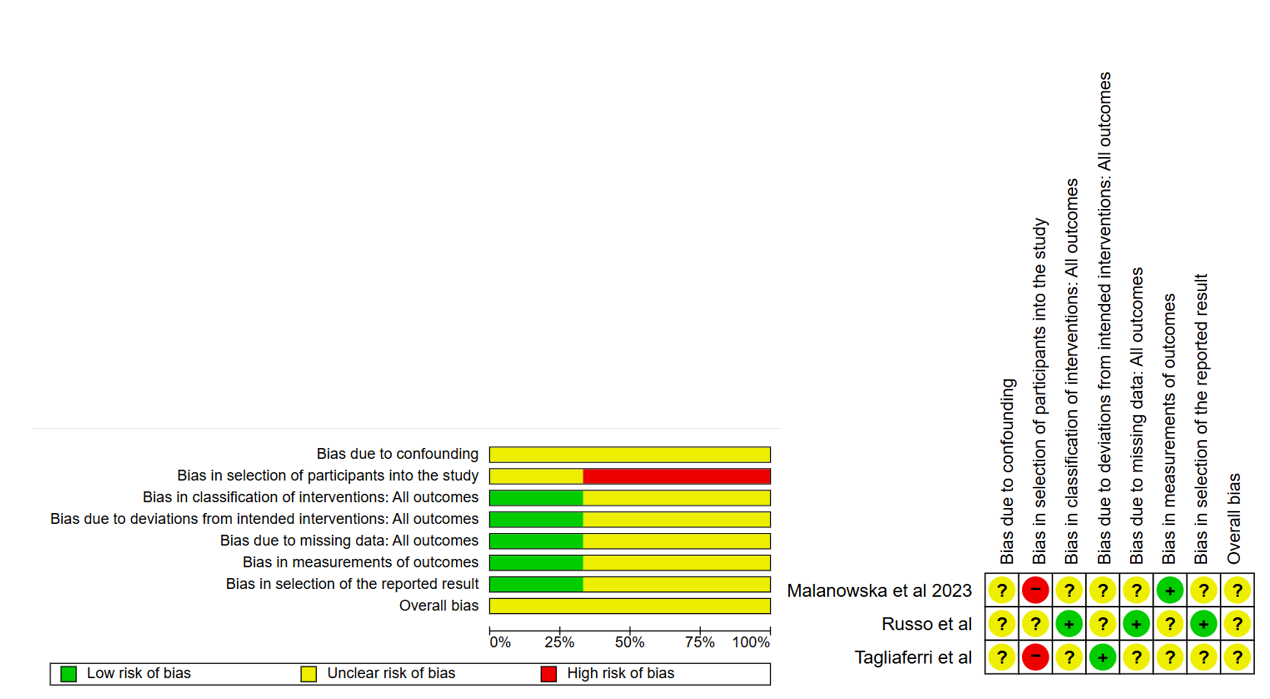

Supplement: Supplementary file 1 — Supplementary file1 (DOCX 861 KB) [file 404_2025_8210_MOESM1_ESM.docx]
